# Complete genome sequence of *Methanothermobacter* sp. DP, a hydrogenotrophic and thermophilic methanogen

**DOI:** 10.1128/mra.00642-23

**Published:** 2023-12-06

**Authors:** Darsha Prabhaharan, Pranav Sasidharan Nair, Hyojung Park, Okkyoung Choi, Byoung-In Sang

**Affiliations:** 1 Department of Chemical Engineering, Hanyang University, Wangsimniro, Seongdong-gu, Seoul, South Korea; 2 Center of Convergence Bioceramic Materials, Korea Institute of Ceramic Engineering and Technology, Osongsaengmyeong 1-ro, Osong-eup, Heungdeok-gu, Cheongju-si, Chungchengbuk-do, South Korea; 3 Eco Lab Center, SK Ecoplant, Jong-ro, Jongno-gu, Seoul, South Korea; 4 Clean-Energy Research Institute, Hanyang University, Wangsimniro, Seongdong-gu, Seoul, South Korea; DOE Joint Genome Institute, Berkeley, California, USA

**Keywords:** thermophilic, hydrogenotrophic, methanogens, genome, *Methanobacterium*

## Abstract

Here, we report the complete genome sequence of the thermophilic hydrogenotrophic methanogen *Methanothermobacter* sp. DP isolated in South Korea from an anaerobic digester fed with cigarette waste. The genome consists of 1,693,285 bp, with 1,772 protein-coding genes and a GC content of 48.8%.

## ANNOUNCEMENT

Wind and solar energy, as renewables, pose challenges for energy storage due to their intermittent nature. Power–to–Gas technology offers efficient storage solutions, utilizing hydrogenotrophic methanogens to convert H_2_ and CO_2_ into CH_4_ suitable for grid injection and an ecofriendly option for energy storage (1, 2).

We isolated *Methanothermobacter* sp. DP, a novel anaerobic, thermophilic methanogen, in a 5 L lab-scale digester. The digester was inoculated with mesophilic (37°C) sludge (1 L) from the Jungrang wastewater treatment plant, in Seoul, South Korea (37.55688 °N and 127.06097 °E), on 30 July 2020. The complete genome sequence has been reported.

Isolation was conducted through sequential subcultures in both liquid (basic anaerobic medium) (3) and solid media (basic anaerobic medium +1.1% gelrite) (4) until single colonies were selected. These colonies were cultivated in liquid media under hydrogenotrophic conditions to assess their methane production. These steps were repeated until a single high-performance methanogen was obtained. *Methanothermobacter* sp. DP grew optimally at 60°C and a pH 7.3 by utilizing CO_2_ and H_2_ (5). After reaching the late exponential phase, DNA was extracted using a FastDNA Spin Kit for Soil (MP Biomedicals, LLC., USA) (6, 7). Primers: 40F (5′-GATTAAGCCATGCAAGTCGAACGA–3’) and 1430R (5′-CTCCTCAAAGAACCCAGATTCGAC-3′) were used for 16S rRNA sequencing and identification. Taxonomic classification was done using MEGA version 6 (8).

The *Methanothermobacter* sp. DP was subjected to whole-genome sequencing at Macrogen Co. Ltd. (Seoul, Republic of Korea). The PacBio sequel system and SMRTbell express template prep kit 2.0 were used to sequence the genome and create a DNA library, respectively (9). Size selection was performed with Agilent Technologies 2100 Bioanalyzer using a DNA 1,000 chip with cut–off range (3,000–50,000 bp). The reads were assembled with SMRTlink version 10.1.0.119588 using Microbial Assembly application (based on HGAP) (10) with the default setting, and the NCBI Prokaryotic Genome Annotation Pipeline was used to annotate gene functions (11).

The filtered reads contained a total of 58,958 reads, which were used for the assembly. The genome comprises 1,693,285 bp with two contigs; contig 1 (1,686,821 bp) and contig 2 (6,464 bp) with a GC content of 48.8% and 43.7%, respectively. The prediction of connected ends suggests circular elements in the genome (12, 13). The genome annotation identified 1,817 genes from both contigs, consisting of 1,772 coding sequences (CDS), 38 tRNAs, and 7 rRNAs (14).

The NCBI BLASTn analysis revealed that *Methanothermobacter* sp. DP contig 1 showed 96% similarity with the *Methanothermobacter marburgensis* strain Marburg (15). Contig 2 showed 99% similarity to the *Methanothermobacter* sp. strain CaT2 (16). *Methanothermobacter* sp. DP comprises 158 CDS associated with energy production. Average Nucleotide Identity of *Methanothermobacter* sp. DP with *M. marburgensis* strain Marburg was 95.5%, below the cut-off value (<95%–96%) indicating a novel species in the genus *Methanothermobacter*.

## Data Availability

The complete genome of *Methanothermobacter* sp. DP is available at NCBI GenBank under the accession number CP113501–CP113502. The sequences are available in the Sequence Read Archive (SRA) under the accession number SRR25498409 and SRR25498410. The BioProject number is PRJNA894335.
